# Verification measurements of the IRMM-1027 and the IAEA large-sized dried (LSD) spikes

**DOI:** 10.1007/s10967-016-5123-8

**Published:** 2016-12-03

**Authors:** R. Jakopič, Y. Aregbe, S. Richter, E. Zuleger, S. Mialle, S. D. Balsley, U. Repinc, J. Hiess

**Affiliations:** 1 European Commission, Joint Research Centre (JRC), Directorate for Nuclear Safety and Security, Standards for Nuclear Safety, Security and Safeguards Unit, Retieseweg 111, 2440 Geel, Belgium; 2 European Commission, Joint Research Centre (JRC), Directorate for Nuclear Safety and Security, Nuclear Safeguards and Forensics Unit, Postfach 2340, 76125 Karlsruhe, Germany; 3 International Atomic Energy Agency, PO Box 100, 1400 Vienna, Austria

**Keywords:** Uranium, Plutonium, Large-sized dried spikes, IDMS, ITV2010

## Abstract

In the frame of the accountancy measurements of the fissile materials, reliable determinations of the plutonium and uranium content in spent nuclear fuel are required to comply with international safeguards agreements. Large-sized dried (LSD) spikes of enriched ^235^U and ^239^Pu for isotope dilution mass spectrometry (IDMS) analysis are routinely applied in reprocessing plants for this purpose. A correct characterisation of these elements is a pre-requirement for achieving high accuracy in IDMS analyses. This paper will present the results of external verification measurements of such LSD spikes performed by the European Commission and the International Atomic Energy Agency.

## Introduction

All states that have signed the treaty on the non-proliferation of nuclear weapons (NPT) officially declare to abandon all efforts to develop nuclear weapon and to conclude safeguards agreements [1, 2]. Nuclear safeguards aims at the verification of the non-diversion of fissile material from its intended and declared (peaceful) use and has the rank of European law. In this context, the determination of the plutonium and uranium contents are required at different stages of a nuclear cycle, in particular in the dissolver solution of irradiated nuclear fuel in a reprocessing plant. Safeguarding reprocessing plants poses a challenge to safeguards authorities because of their size, high material throughput and the requirement for high level of detection probability of diverted material.

Isotope dilution analysis (IDA) is widely applied as a reliable analytical technique for measurements of uranium and plutonium in dissolved nuclear fuel and for achieving high accuracy results [3–8]. In IDA, the amount of an element (e.g. U and Pu) in the sample is determined on the basis of an addition of a known amount of the same element whose isotopic composition deliberately differs from that of the sample (called a spike). By measuring the change in the isotopic composition of the sample-spike mixture (a blend) by isotope mass spectrometry, the unknown amount of the element in the sample can be calculated [7, 9]. Highly enriched ^233^U and ^242^Pu (or even ^244^Pu) spikes are commonly applied in IDA of a nuclear material when the concentration of the sample being measured is low and suitable for handling in a radiochemical laboratory. However, due to high concentrations of uranium, plutonium and other fission products in the dissolver solution, dilution steps would be required to levels suitable for these spikes under typical glove-box conditions. The dilution steps required introduce an additional uncertainty into the whole measurement procedure.

The use of much larger spikes containing ^239^Pu and ^235^U isotopes applied directly to the dissolved nuclear material was already proposed about 30 years ago to circumvent the need for dilution. They contain milligram rather than microgram quantities of nuclear material and are in the dried form, the so-called large-sized dried (LSD) spikes. The main advantage of using LSD spikes is that the dilution of a sample of a dissolved nuclear fuel solution is no longer required, therefore simplifying the weighing process and reducing the overall uncertainty of the measured amounts of plutonium and uranium [5, 10, 11]. Highly enriched and pure certified reference metals of ^239^Pu, ^238^U and ^235^U are used as source material for the preparation of these spikes.

At present the LSD spikes are produced and certified by the Joint Research Centre in Geel (JRC-Geel) [12, 13]. These spikes, commonly known as the IRMM-1027 series are produced annually in batches of about 1200 units to fulfil the demands for fissile material control by safeguards authorities and plant operators. They are applied at the on-site laboratories of the two European reprocessing plants at Sellafield (UK) and La Hague (FR) and in industry (Sellafield Limited, Japan Nuclear Fuel Limited). Similar spikes are produced on somewhat smaller scale by the International Atomic Energy Agency (IAEA) and applied at the on-site laboratory of the Rokkasho reprocessing plant (RRP) in Japan. Solid spikes containing ^235^U and ^239^Pu have been successfully utilised for safeguards inspections and in accountability analysis for operators at the reprocessing plants [10, 12, 14–16]. The extensive use of these spikes over the past years has demonstrated that IDMS, applying properly characterised spikes routinely provides accurate results with low uncertainties as required by safeguards authorities. The international target values (ITV2010) are uncertainties to be considered when judging the reliability of the measurement results [17]. They represent the estimates of the state of the practice and should be achievable under routine measurement conditions in a typical industrial laboratory or during actual inspections [17].

For many years the JRC-Geel, Belgium and the IAEA have been producing LSD spikes for this purpose. In 2008, the IAEA proposed to the JRC of the European Commission (EC) a Support programme task on “verification of mixed U/Pu spikes” [18]. Since then mutual verification measurements of the produced LSD spikes are carried out by the JRC-Geel, Belgium, and the IAEA as LSD spike producers and the JRC-Karlsruhe, Germany as user of LSD spikes for operating the European Safeguards On-site Laboratories. These independent verification measurements are important for the spike suppliers in view of providing high quality spikes to the safeguards community. At the same time they can be helpful to identify any potential bias that may exist within a laboratory’s measurement systems, during the spike preparation and usage. In this paper the results of the verification measurements of 7 different batches of LSD spikes will be presented and discussed: IRMM-1027o, IRMM-1027p, IRMM-1027q and IRMM-1027r produced by JRC-Geel and SAL-24, SAL-25 and SAL-26 produced by the IAEA. The results will be compared to the assigned values and the respective ITV2010.

## Experimental

### Preparation and characterisation of the LSD spikes

IRMM-1027 LSD spikes are produced in compliance with the ISO Guide 34 [19] by dissolving high purity and highly enriched plutonium and uranium certified reference metals in acid, dispensing the solution into individual penicillin vials and drying. The dried spikes are treated with cellulose acetate butyrate (CAB) to fix the spike material at the bottom of the vial during shipment and storage [20]. The starting materials of uranium and plutonium are blended to give a fixed ratio of uranium to plutonium of approximately 30:1. This ratio was selected at the time with users as being suitable for the measurement of the wide range of typical dissolved fuel. Each individual unit of IRMM-1027 spike contains about 2 mg plutonium and 55 mg uranium. The uranium and plutonium components in the spike are enriched to about 20% in ^235^U and 98% in ^239^Pu, respectively. The IRMM-1027 spikes are certified for the mass of ^235^U, ^238^U and ^239^Pu per unit and the *n*(^234^U)/*n*(^238^U), *n*(^235^U)/*n*(^238^U), *n*(^240^Pu)/*n*(^239^Pu), *n*(^241^Pu)/*n*(^239^Pu), and *n*(^242^Pu)/*n*(^239^Pu) amount ratios. Values of the U and Pu isotope mass fractions, amount contents and the *n*(^236^U)/*n*(^238^U) and *n*(^238^Pu)/*n*(^239^Pu) amount ratios are provided in the material`s certificate as additional information. Details on the preparation and certification can be found in the certification reports [21–24].

The IAEA LSD spikes are prepared for IAEA internal use only in a similar way; however no organic additive is applied on the dried spikes for stabilisation. Drying of the nitrate solution in the vials is performed at a temperature of 125–135 °C in order to produce a glassy and strongly adherent deposit of uranyl and plutonium nitrate [25]. The ratio of uranium to plutonium in the spike, the unit size and the ^235^U enrichment vary between different batches. The spikes prepared by IAEA-NML are not certified for the mass of plutonium or uranium, nor the isotopic composition per vial as the IRMM-1027 series are. Instead each batch of the LSD spike solution is prepared by mixing U and Pu stock solutions, which are gravimetrically prepared from certified reference materials (CRMs). Stock solutions are verified for their U and Pu mass fraction and isotopic composition using independent measurement techniques (Davies and Gray titration and IDMS for U, controlled potential coulometry and IDMS for Pu). After aliquoting the spike solution into individual penicillin vials and drying, randomly selected vials are characterised by IDMS. Characteristics of the LSD spikes from the JRC and the IAEA are summarised in Table 1.

**Table 1:** Characteristics of the IAEA SAL and IRMM-1027 LSD spikes

Spike batch	IAEA SAL-24	IAEA SAL-25	IAEA SAL-26	IRMM-1027o	IRMM-1027p	IRMM-1027q	IRMM-1027r
U source^a^	CRM 116	CRM 112-A, CRM 116	CRM 116	EC NRM 101, CRM 116	EC NRM 101, CRM 116	EC NRM 101, CRM 116	EC NRM 101, CRM 116-A
Pu source^a^	MP2	MP2	CRM 126-A	MP2	MP2	MP2	MP2
Dissolution	HNO_3_ (conc.), HF (0.01 mol/L), heating			HNO_3_ (8 mol/L), traces HF, heating 60–70 °C			HCl (conc.), HNO_3_ (8 mol/L), heating 60–70 °C)
Dispensing	Manual			Automated system			
Drying	125–135 °C			60 °C			
Coating	No coating			CAB-35^b^ (drying 45–50 °C)			
Units prepared	360	340	358	1215	1186	1126	1151
U:Pu ratio	7	18	5	30	29	29	29
^235^U enrichment^c^ (%)	93	20	93	19	17	18	19
^239^Pu enrichment^c^ (%)	98	98	97	98	98	98	98
Unit size (mg)	U: 11 Pu: 1.6	U: 31 Pu: 1.8	U: 11 Pu: 2	U: 54 Pu: 1.8	U: 54 Pu: 1.8	U: 55 Pu: 1.9	U: 55 Pu: 1.9

^a^CRM 116, CRM 116-A, CRM 112-A and CRM 126 (NBL, USA), MP2 (CEA/CETAMA, France), EC NRM 101 (JRC, Belgium)

^b^CAB-35: cellulose acetate butyrate with relative butyryl mass fraction of 34–39 [expressed as *m*(butyryl)/*m*(CAB)] [20]

^c^Relative isotope mass fraction [expressed as *m*(^235^U)/*m*(U) and *m*(^239^Pu)/*m*(Pu)]

### Verification measurements

Several units of the chosen batch were measured for the uranium and plutonium content and isotope amount ratios by three laboratories: the JRC-Karlsruhe (Lab A), JRC-Geel (Lab B) and IAEA-NML (Lab C). In the case of IAEA SAL-25, samples were also analysed by the Nuclear Material and Control Centre, Tokai Safeguards Center in Japan (Lab D). Units of the IRMM-1027 series were randomly selected from the whole batch by a stratified sampling method. Multi-collector thermal ionisation mass spectrometry (MC-TIMS) was used in all cases to measure the Pu and U isotope amount ratios. The isotopic measurements were performed in total evaporation (TE) mode, which is a frequently applied technique to minimise the mass fractionation effects [26–29]. Prior to the measurement a chemical separation of the uranium and plutonium was performed (see Table 2). Various CRMs were used as spikes for IDMS analysis to determine the U and Pu amount content, either as liquid spikes or in the form of oxides and metals. In some cases the spike solutions were prepared from in-house materials. Plutonium measurements were corrected for radiometric decay since the certification date of the starting reference materials. Details of various spikes, chemical procedures and measurement protocols are summarised in Table 2 and described in [30].

**Table 2:** Spikes and procedures for the analysis of the IRMM-1027 and the IAEA SAL LSD spikes

Laboratory	IAEA-NML	JRC-Karlsruhe [30]	JRC-Geel [21–24]
U spike for IDMS	^235^U (CRM 116)^a^ ^235^U (UB3_SP)^b^ ^238^U (CRM 112-A)^c^ ^233^U/^242^Pu (IRMM-046c)^d^	^238^U (EC-110)	^233^U/^242^Pu (IRMM-046c)^d^ ^233^U/^242^Pu (IRMM-046b)^e^
Pu spike for IDMS	^240^Pu (PR_SP3 in-house)^f^ ^242^Pu (KRI-RM1 662)^g^ (KRI-RM2 662)^h^ ^233^U/^242^Pu (IRMM-046c)^d^ ^242^Pu (IRMM-049d)^i^	^240^Pu (SM4 in-house) calibrated with MP2	^233^U/^242^Pu (IRMM-046c)^d^ ^233^U/^242^Pu (IRMM-046b)^e^
External QC (PT)	EQRAIN^j^	EQRAIN^j^	EQRAIN^j^
Spiking/weighing	Single weighing	Double weighing method	Substitution weighing
U/Pu separation	TOPO resin: Valence adjustment:/ Sample loading: 3 M HNO_3_ Pu elution: formic/ascorbic acid U elution: ammonium carbamate solution	UTEVA resin: Valence adjustment: H_2_O_2_ Sample loading: 6 M HNO_3_ Pu elution: hydroxylamine/ascorbic acid in 2 M HNO_3_ U elution: ammonium oxalate solution	Anion exchange: Valence adjustment: FeCl_2_/NH_4_OCl/NaNO_2_ Sample loading: 8 M HNO_3_ U elution: 8 M HNO_3_ Pu elution: 0.35 M HNO_3_
Mass spectrometer	Triton TIMS	MAT 262^k^ Triton TIMS^l^	Triton TIMS
Mass bias correction	None^m^	None^n^	IRMM-290/A3 for Pu^o^ IRMM-074/10 for U^p^
Filament	Rhenium (Re) for ionisation filament Tungsten (W) for evaporation filament	Rhenium (Re) for ionisation filament Tungsten (W) for evaporation filament	Rhenium (Re)
Sample loading for TIMS	U: 500 ng, Pu: 100 ng	U: 100 ng Pu: 10 ng	U: 100 ng Pu: 50 ng
Number of replicate filament measurements from a single LSD vial	1–2	2	3–4
Measurement method	Total evaporation	Total evaporation	Total evaporation

^a^Used for IAEA SAL-25, IRMM-1027o, IRMM-1027p and IRMM-1027q

^b^Used for IRMM-1027r

^c^Used for IAEA SAL-24, IAEA SAL-26 and IRMM-1027r

^d^Used for IRMM-1027q, IAEA SAL-24, IAEA SAL-25 and IAEA SAL-26

^e^Used for IRMM-1027o, IRMM-1027p, IRMM-1027q and IRMM-1027r

^f^Used for IAEA SAL-24, IAEA SAL-25, IAEA SAL-26 and IRMM-1027o

^g^Used for IRMM-1027p and IRMM-1027q

^h^Used for IRMM-1027r

^i^Used for IRMM-1027r

^j^EQRAIN (evaluation de la Qualité du Résultat d’Analyse dans l’Industrie Nucléaire)

^k^Used for IAEA SAL-24, IAEA SAL-25, IAEA SAL-26, IRMM-1027o and IRMM-1027p

^l^Used for IRMM-1027q and IRMM-1027r

^m^Quality control with CRM-136, CRM-137, CRM-138, CRM-112a, CRM U-500, CRM U-930

^n^Quality control with IRMM-199, IRMM-290F and in-house RM

^o^Quality control with IRMM-290/G3

^p^Quality control with IRMM-074/2/3

Using the spike, the U and Pu content in LSD spikes can be determined following the general IDMS equation (Eq. 1) or similar, depending on the spike and procedures applied in the laboratories.

1 $$c_{\text{x}} = c_{\text{y}} \frac{{m_{\text{y}} }}{{m_{x} }}\frac{{R_{\text{y}} - R_{\text{b}} }}{{R_{\text{b}} - R_{\text{x}} }}\frac{{\mathop \sum \nolimits (R_{i} )_{\text{x}} }}{{\mathop \sum \nolimits (R_{i} )_{\text{y}} }}$$

where *c*_y_ is the element amount content of the spike, *m*_x_ is the mass of the sample, *m*_y_ that of the spike, *R*_x_, *R*_y_ and *R*_b_ are the isotope amount ratios of the sample, the spike and the blend, respectively, $\mathop \sum \nolimits (R_{i} )_{\text{x}}$ and $\mathop \sum \nolimits (R_{i} )_{\text{y}}$ are the sums of all isotope amount ratios in sample and in spike, respectively.

## Results and discussion

### IAEA LSD spikes

**Fig. 1:**
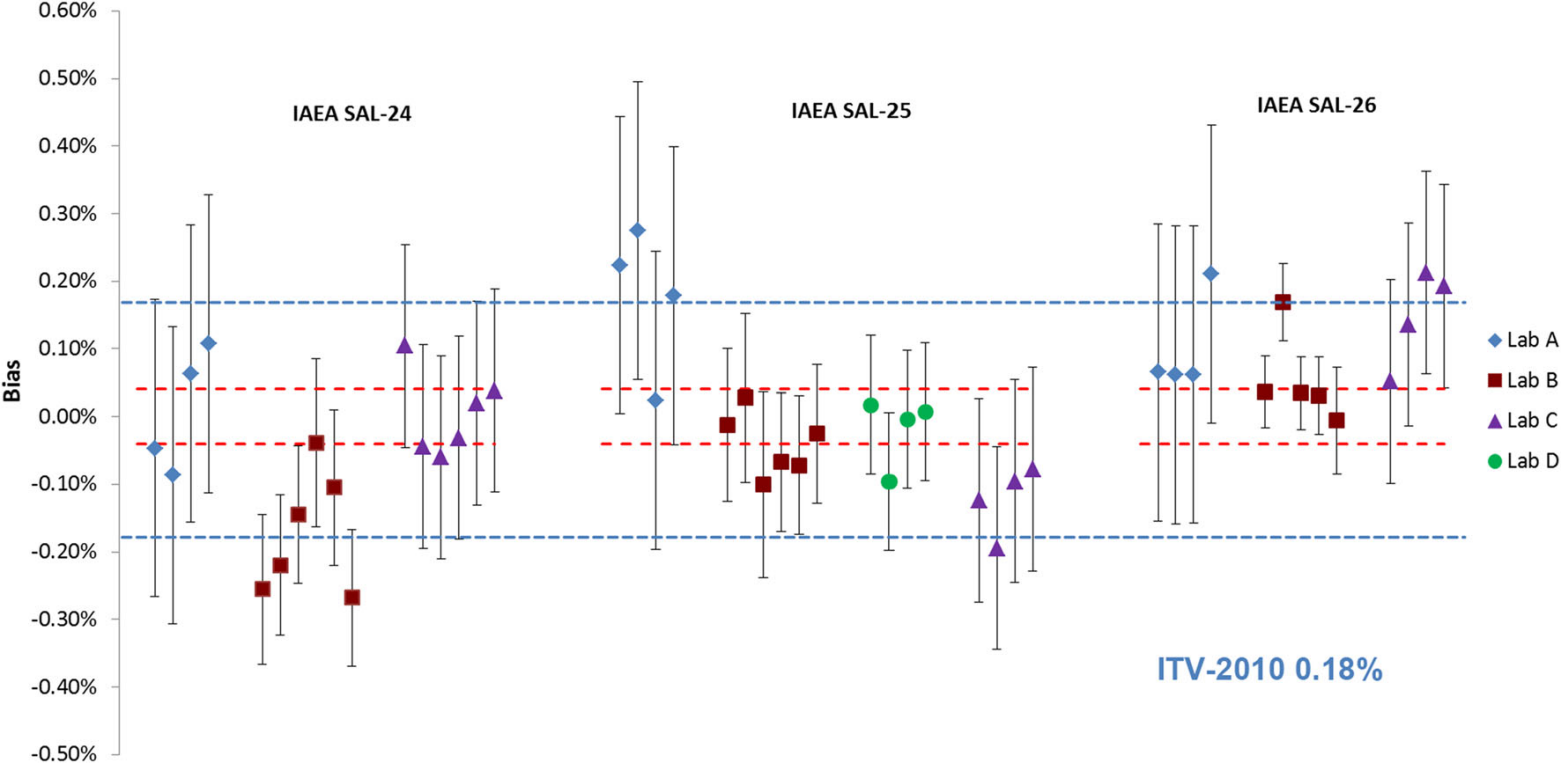
Results of the verification measurements for the Pu amount content in IAEA SAL-24, IAEA SAL-25 and IAEA SAL-26 expressed as the relative difference (bias) from the assigned value. *Error bars* show the relative expanded uncertainty of the reported measurement result. *Red dotted lines* show the relative expanded uncertainty (*k* = 2) of the assigned value and the *blue dotted lines* the respective ITV2010 value (expressed as the relative combined standard uncertainty). (Color figure online)

**Fig. 2:**
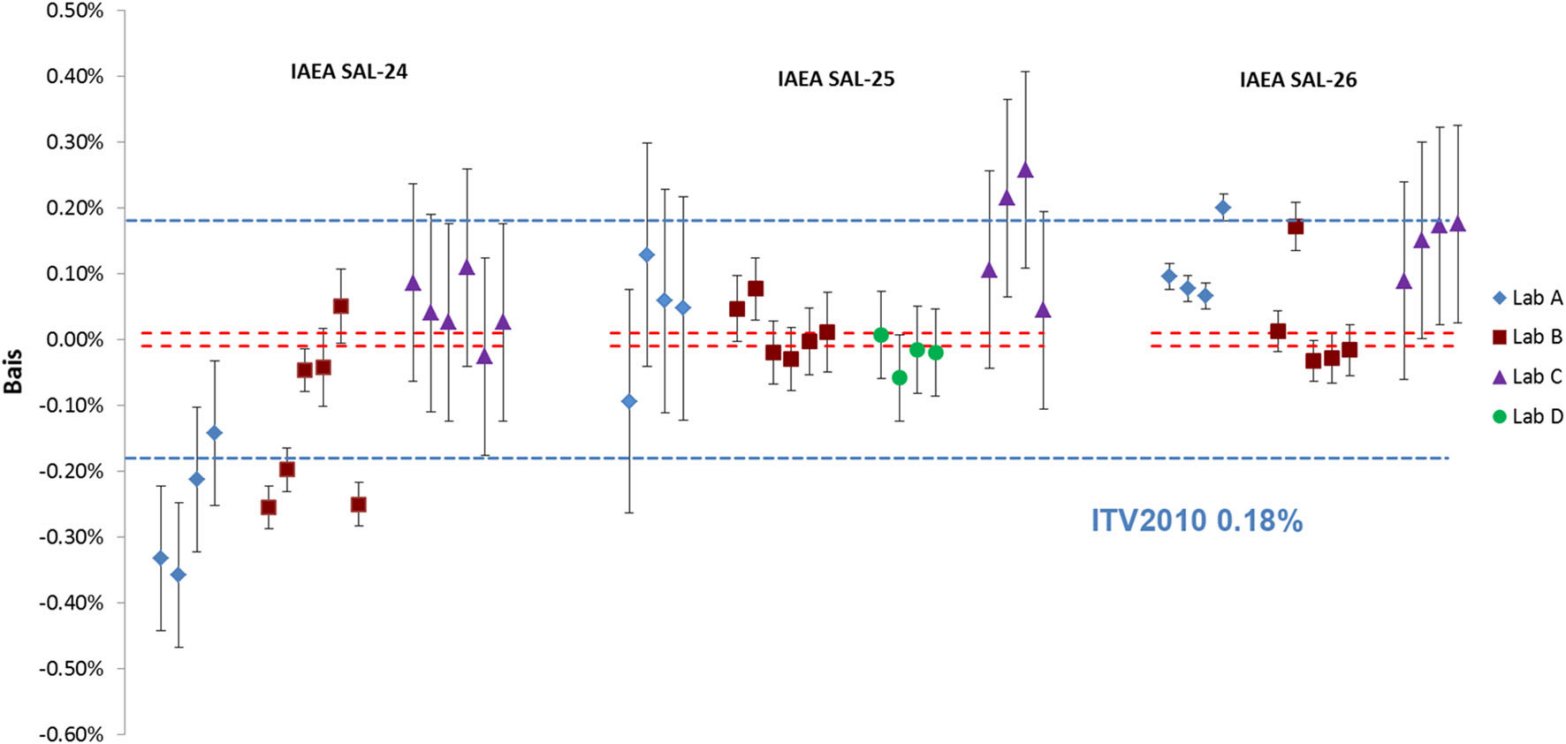
Results of the verification measurements for the U amount content in IAEA SAL-24, IAEA SAL-25 and IAEA SAL-26 expressed as the relative difference (bias) from the assigned value. *Error bars* show the relative expanded uncertainty of the reported measurement result. *Red dotted lines* show the relative expanded uncertainty (*k* = 2) of the assigned value and the *blue dotted line* the respective ITV2010 value (expressed as the relative combined standard uncertainty). (Color figure online)

Results of the plutonium and uranium amount content measurements for IAEA SAL-24, IAEA SAL-25 and IAEA SAL-26 are shown in Figs. 1 and 2. Each data point represents an independent measurement result of a selected unit of LSD spike (e.g. chemical treatment, replicate measurements). Details about spikes used among all laboratories for all batches are summarised in Table 2. The individual results are expressed as the relative difference (bias in %) from the assigned value of the U/Pu solution prepared from the CRM metals. The relative expanded uncertainties (*k* = 2) of the assigned value for uranium is 0.01% and for plutonium 0.04%.

For the majority of the results for Pu content, the biases were smaller than the target uncertainty value of 0.18% for glove-box conditions [17] as shown in Fig. 1, except for some of the individual results. In some cases, the reported results did not agree with the assigned value within measurement uncertainty. Similar trends can be observed for the U amount content in Fig. 2. Also here for the majority of the U results, the biases are smaller than the respective ITV2010 value, however fewer results agreed with the assigned value within the measurement uncertainty. The biases were in the same order of magnitude as for the Pu results, but the uncertainty of the assigned value for the U amount content was much smaller. This is due to inherent characteristics of the uranium CRM metals. It can also be observed that laboratories were consistent with reporting the measurement uncertainties except for the U results in IAEA SAL-26, where smaller uncertainties were reported by the laboratory A compared to IAEA SAL-24 and IAEA SAL-25. Some differences were observed in the reported measurement uncertainties among the laboratories. This is due to different approaches used for uncertainty estimation by the laboratories. Laboratories A and B provided the full uncertainty budget according to GUM [31–33], taking all available sources of uncertainty into account (e.g. weighing, spike reference materials, measurement repeatability, etc.). Laboratory C estimated the measurement uncertainty as the random component of ITV2010 for IDMS (glove-box conditions) as expected performance of a laboratory carrying out safeguards verification activities [17].

Interestingly, the IDMS associated with high precision MC-TIMS TE method reveals systematically different results for Pu and U amount content, within all three LSD spike batches from laboratory to laboratory. This could be the result of differences in the spikes used by the different laboratories, however additional measurements would be required to confirm this observation.

**Fig. 3:**
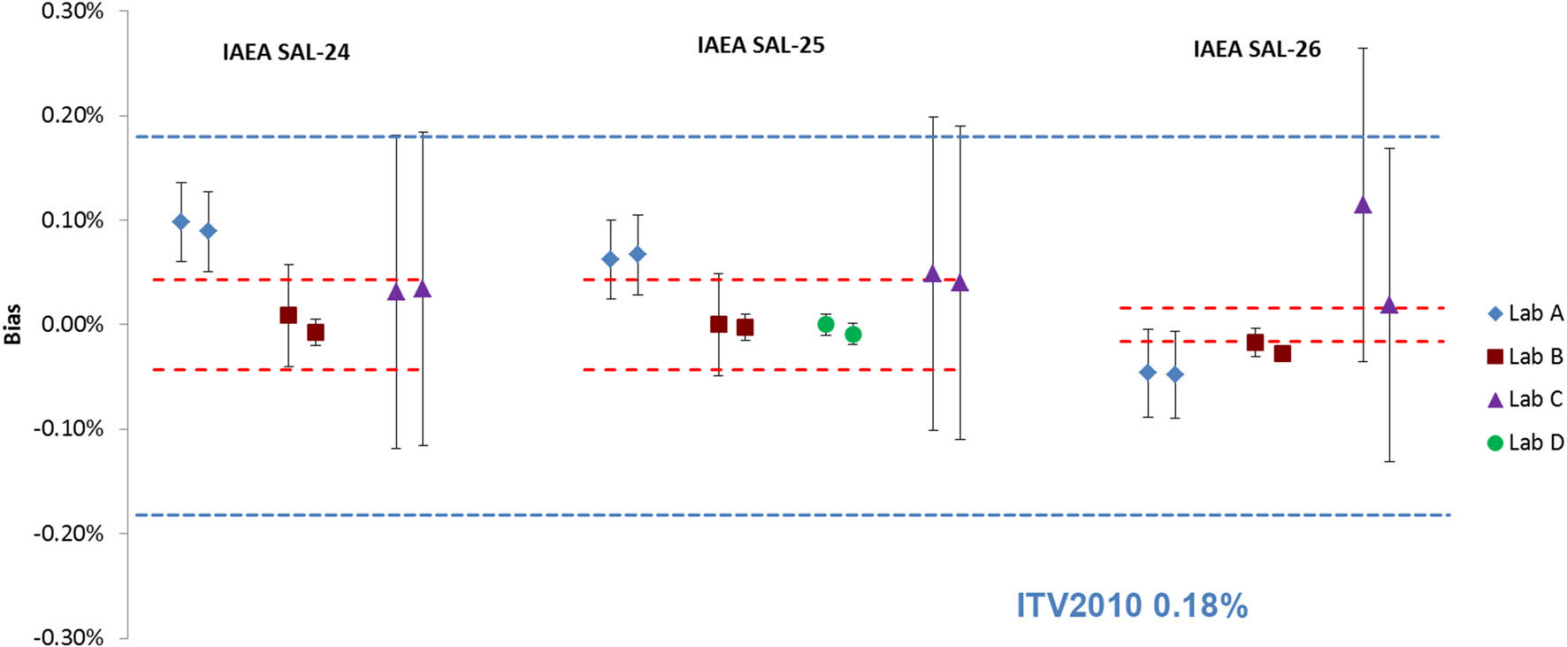
Results of the verification measurements of the *n*(^240^Pu)/*n*(^239^Pu) ratio in IAEA SAL-24, IAEA SAL-25 and IAEA SAL-26 expressed as the relative difference (bias) from the assigned value. *Error bars* show the relative expanded uncertainty of the reported measurement result. *Red dotted lines* show the relative expanded uncertainty (*k* = 2) of the assigned value and the *blue dotted line* the respective ITV2010 value (expressed as the relative combined standard uncertainty). (Color figure online)

**Fig. 4:**
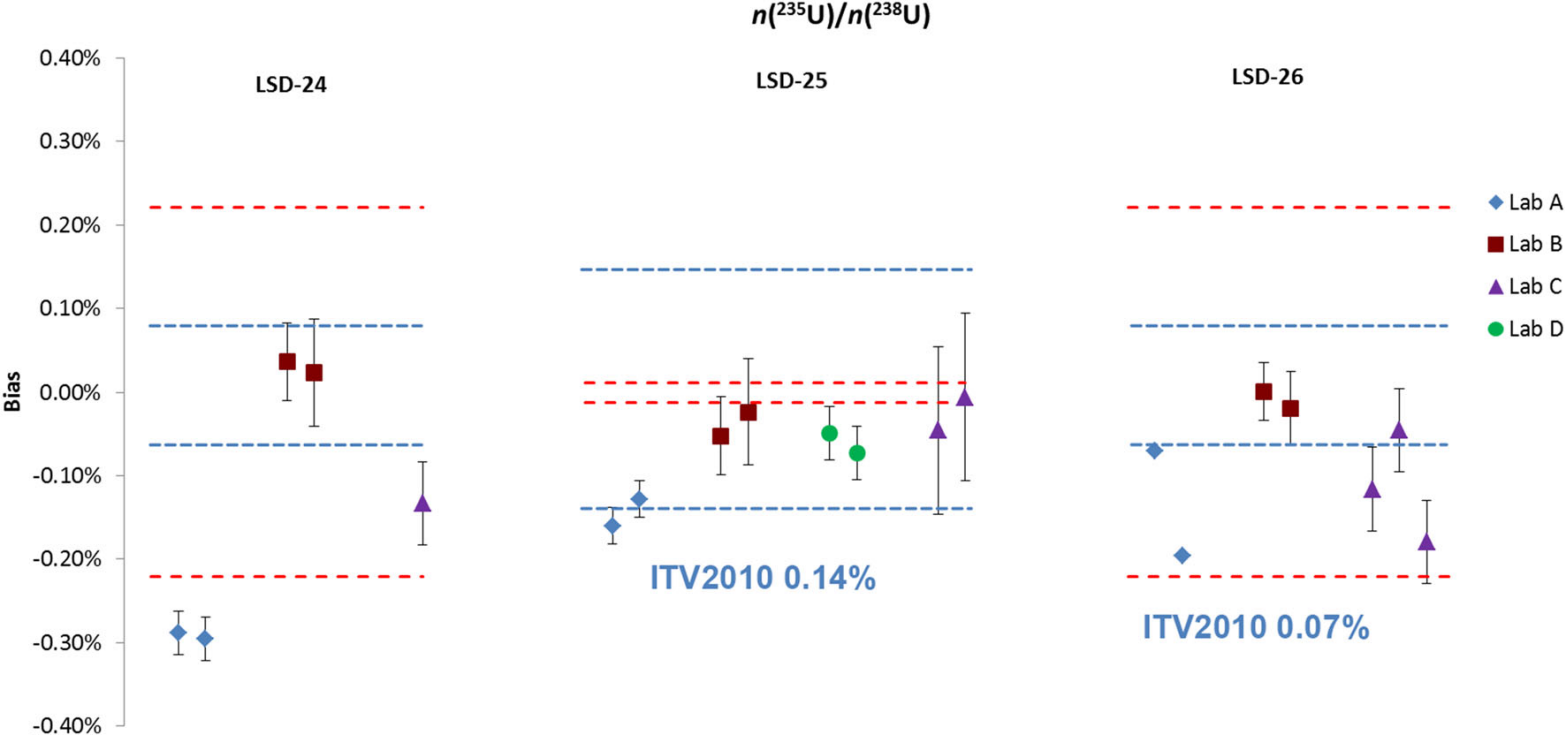
Results of the verification measurements of the *n*(^235^U)/*n*(^238^U) ratio in IAEA SAL-24, IAEA SAL-25 and IAEA SAL-26 expressed as the relative difference (bias) from the assigned value. *Error bars* show the relative expanded uncertainty of the reported measurement result. *Red dotted lines* show the relative expanded uncertainty (*k* = 2) of the assigned value and the *blue dotted line* the respective ITV2010 value (expressed as the relative combined standard uncertainty). The ITV2010 value for ^235^U abundance were used for the purpose of this study. (Color figure online)

Results of the *n*(^240^Pu)/*n*(^239^Pu) and *n*(^235^U)/*n*(^238^U) amount ratios in IAEA SAL-24, IAEA SAL-25 and IAEA SAL-26 are shown in Figs. 3 and 4, respectively. The majority of the *n*(^240^Pu)/*n*(^239^Pu) results are in agreement with the assigned value and within the respective ITV2010. There are no ITV2010 values for *n*(^235^U)/*n*(^238^U) amount ratios, instead the values for ^235^U abundance were used as alternative for the purpose of this study. Some differences were observed in the reported measurement uncertainties among the laboratories.

In general, larger differences among laboratories were observed for the *n*(^235^U)/*n*(^238^U) ratio results. With the exception of the IAEA SAL-25 (low enriched U), larger differences were observed in IAEA SAL-24 and IAEA SAL-26 due to complexity of analysis (high enriched U material is more affected by cross contamination). The assigned values for the IAEA LSD spikes and associated measurement uncertainty are calculated using a formula taking all available sources of uncertainties into account. IAEA SAL-24 and IAEA SAL-26 were prepared solely from CRM 116 (see Table 1), which has no certified value for the *n*(^235^U)/*n*(^238^U) ratio but is certified only for the ^235^U wt% abundance. The dominant factor to the combined uncertainty for the *n*(^235^U)/*n*(^238^U) ratio is therefore the ^238^U isotope abundance and associated measurement uncertainty that was derived experimentally in 1984. IAEA SAL–25 was prepared from mixture of CRM 112-A and CRM 116 where only uncertainty of source material as provided on the certificates were taken into account.

### IRMM-1027 LSD spikes

**Fig. 5:**
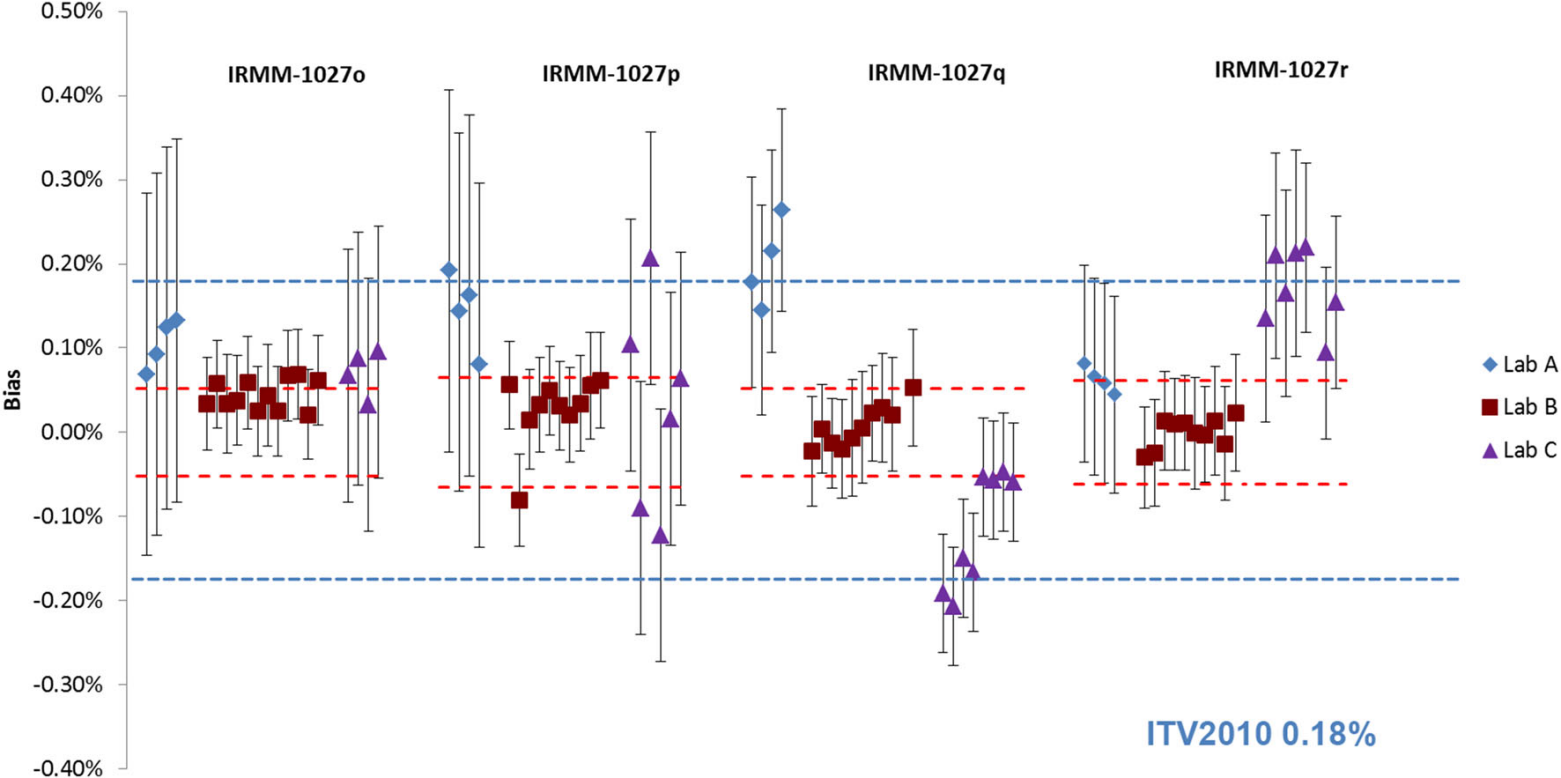
Results of the verification measurements of the Pu amount content in IRMM-1027o, IRMM-1027p, IRMM-1027q and IRMM-1027r expressed as the relative difference (bias) from the certified value. *Error bars* show the relative expanded uncertainty of the reported measurement result. *Red dotted lines* show the relative expanded uncertainty (*k* = 2) of the certified value and the *blue dotted line* the respective ITV2010 value (expressed as the relative combined standard uncertainty). (Color figure online)

**Fig. 6:**
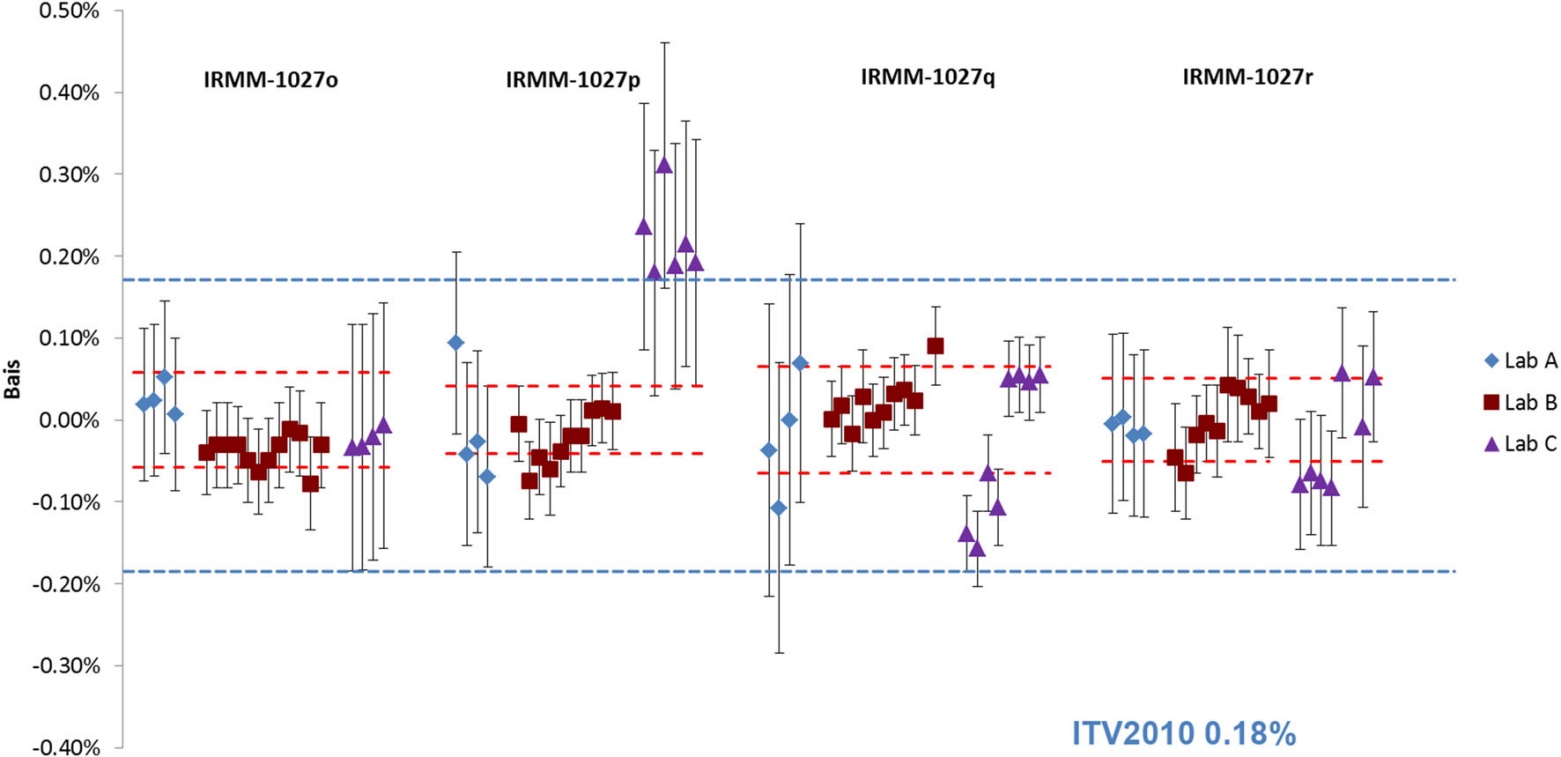
Results of the verification measurements of the U content in IRMM-1027o, IRMM-1027p, IRMM-1027q and IRMM-1027r expressed as the relative difference (bias) from the certified value. *Error bars* show the relative expanded uncertainty of the reported measurement result. *Red dotted lines* show the relative expanded uncertainty (*k* = 2) of the certified value and the *blue dotted line* the respective ITV2010 value (expressed as the relative combined standard uncertainty). (Color figure online)

Results for the Pu and U amount content in the IRMM-1027o, IRMM-1027p, IRMM-1027q and IRMM-1027r are shown in Figs. 5 and 6, respectively. Similar to the IAEA LSD spikes, the certified values were based on the gravimetric preparation from CRM metals. The exceptions are the Pu amount contents in IRMM-1027q and IRMM-1027r, where due to a technical problem during the preparation, the assignment of the certified values was established by IDMS using TIMS [23, 24]. The relative expanded uncertainty (*k* = 2) of the certified values are in the range of 0.04–0.07% for both the U and Pu content. The certified values of the IRMM-1027 LSD spikes have somewhat larger uncertainties compared to the IAEA LSD spikes due to an additional uncertainty component from the homogeneity assessment [19, 21–24].

It can be seen from Fig. 5 that the reported results for the Pu content in IRMM-1027o, IRMM-1027p and IRMM-1027r agreed with the certified value within the measurement uncertainty. The differences from the certified value were smaller than the target values. On the other hand, agreement for the IRMM-1027q was achieved, only for some of the results reported by laboratories B and C. The same grouping of the U results was observed for laboratory C (Fig. 6). Detailed examination of the results from laboratory C showed the use of two different spikes for the U and Pu IDMS analysis of IRMM-1027q, and spike that produced systematically negative bias was a mixed U/Pu spike.

A good agreement was obtained for the U results shown in Fig. 6, except for the IRMM-1027p results reported by the laboratory C. Systematically higher or lower results could be an artefact of the chosen spike, whereas a large spread in measurement results could indicate some problems in measurement repeatability and/or reproducibility. In general, the majority of the reported results for the U and Pu content were within the ITV2010 target values.

**Fig. 7:**
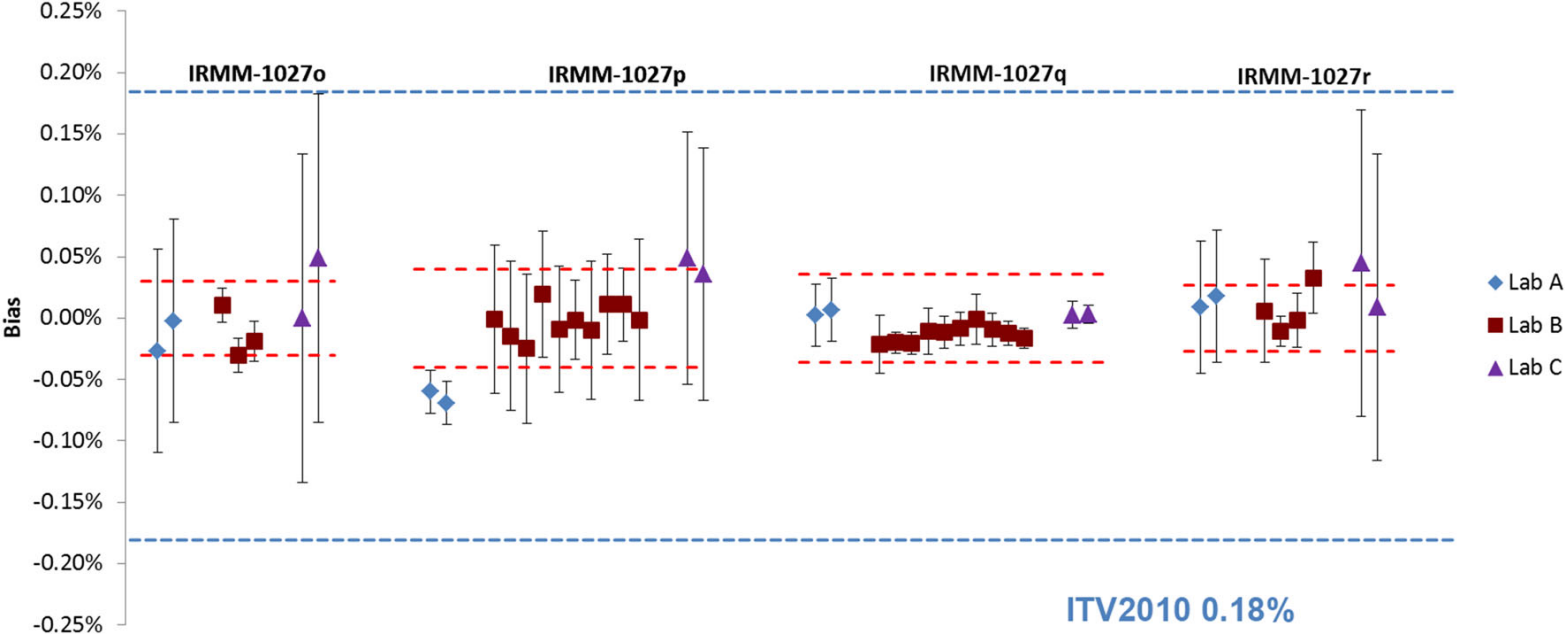
Results of the verification measurements of the *n*(^240^Pu)/*n*(^239^Pu) ratio in IRMM-1027o, IRMM-1027p, IRMM-1027q and IRMM-1027r expressed as the relative difference (bias) from the certified value. *Error bars* show the relative expanded uncertainty of the reported measurement result. *Red dotted lines* show the relative expanded uncertainty (*k* = 2) of the certified value and the *blue dotted line* the respective ITV2010 value (expressed as the relative combined standard uncertainty). (Color figure online)

**Fig. 8:**
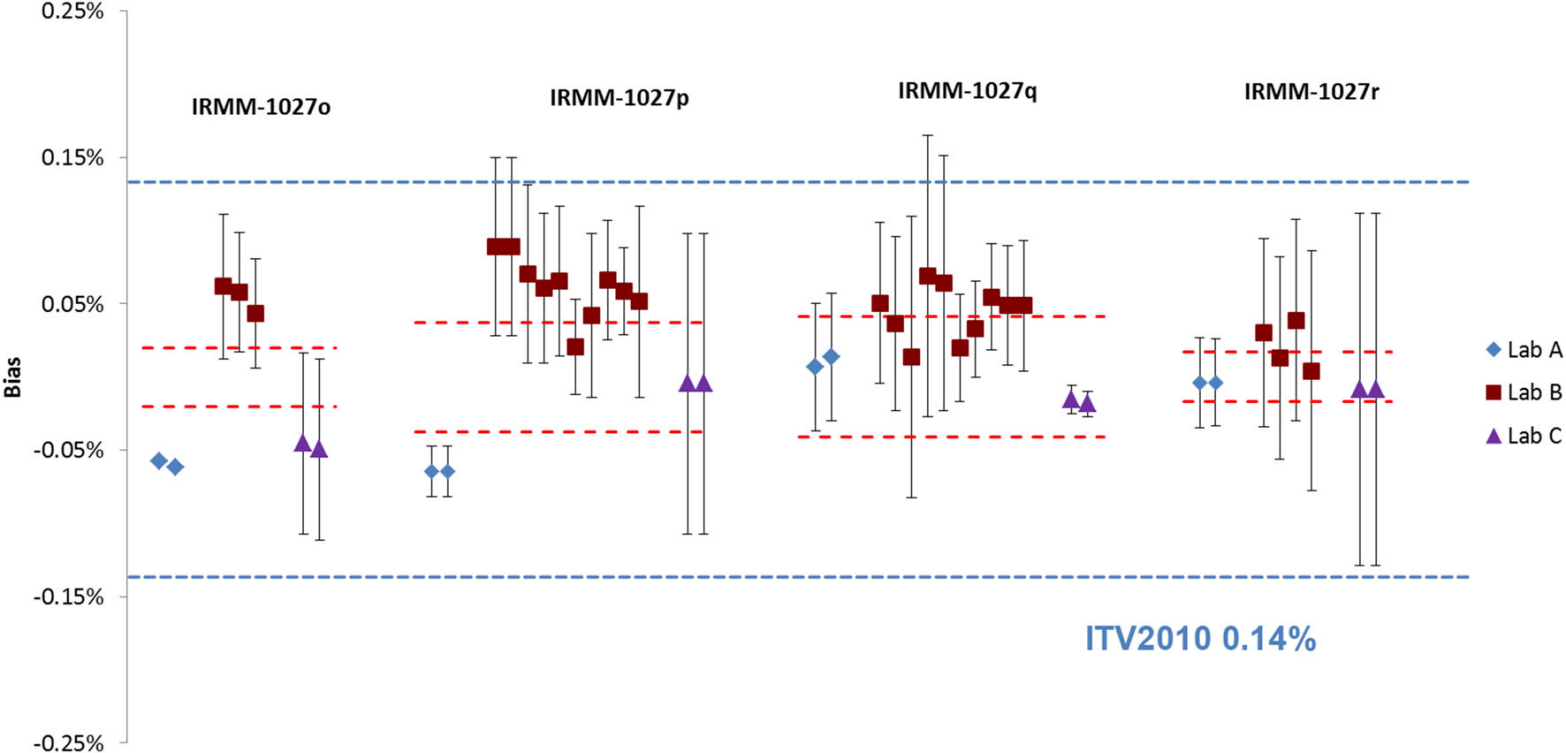
Results of the verification measurements of the *n*(^235^U)/*n*(^238^U) ratio in IRMM-1027o, IRMM-1027p, IRMM-1027q and IRMM-1027r expressed as the relative difference (bias) from the certified value. *Error bars* show the relative expanded uncertainty of the reported measurement result. *Red dotted lines* show the relative expanded uncertainty (*k* = 2) of the certified value and the *blue dotted line* the respective ITV2010 value (expressed as the relative combined standard uncertainty). The ITV2010 value for ^235^U abundance were used for the purpose of this study. (Color figure online)

The results of the *n*(^240^Pu)/*n*(^239^Pu) and *n*(^235^U)/*n*(^238^U) amount ratios in the IRMM-1027 LSD spikes are shown in Figs. 7 and 8, respectively. All the *n*(^240^Pu)/*n*(^239^Pu) results are in agreement with the certified value within the measurement uncertainty, except for the laboratory A in IRMM-1027p. The majority of the *n*(^235^U)/*n*(^238^U) results also agreed with the certified value, except for the IRMM-1027o and IRMM-1027p results reported by the laboratory A. This disagreement was due to very low measurement uncertainties reported by this laboratory. Systematically higher results compared to the certified value were observed for the laboratory B for all IRMM-1027 LSD samples.

## Conclusions and outlook

The results presented in this paper have shown that transparent mutual verification measurements of the IRMM-1027 series and the IAEA LSD spikes in the frame of the EC Support Programme to the IAEA are valuable for the reference material producers and the LSD spike users. The three laboratories could confirm that the ITV2010 values are achievable target parameters and fit for purpose. The obtained results confirm that IDMS using LSD spike is a reliable method providing high accuracy measurement results, which are needed to draw nuclear safeguards conclusions. Another important benefit of the exchange is the opportunity for the laboratories to identify problems and potential areas of improvement. For example, since all laboratories use IDMS for determination of U and Pu amount contents in the LSD spikes, the exchange offers a good opportunity to evaluate and better understand the sources of discrepancies that may be intrinsic to the spike materials used in this study themselves. However, it can also be seen from this study that the same spike used for IDMS analysis of different LSD spikes gives for one laboratory IDMS results in agreement with the assigned values and for other laboratory results in disagreement. This brings us back to the two incentives of this paper as already emphasised in the introduction that external verification is not only a beneficial tool to demonstrate confidence in certified/assigned values of LSD but also helps to identify and resolve any potential measurement problems that might exist within a laboratory.
